# Sharing of retracted COVID-19 articles: an altmetric study

**DOI:** 10.5195/jmla.2022.1269

**Published:** 2022-01-01

**Authors:** Amrollah Shamsi, Brady Daniel Lund, Shohreh SeyyedHosseini

**Affiliations:** 1 shamsiamrollah@gmail.com, Independent Research, Bushehr, Iran; 2 blund2@g.emporia.edu, School of Library and Information Management, Emporia State University, Emporia, KS; 3 tanin64@gmail.com, Knowledge and Information Science, Bushehr University of Medical Sciences, Bushehr, Iran

**Keywords:** COVID-19, retractions, altmetrics, articles, social media

## Abstract

**Objective:**

This study examines the extent to which retracted articles pertaining to COVID-19 have been shared via social and mass media based on altmetric scores.

**Methods:**

Seventy-one retracted articles related to COVID-19 were identified from relevant databases, of which thirty-nine had an Altmetric Attention Score obtained using the Altmetrics Bookmarklet. Data extracted from the articles include overall attention score and demographics of sharers (e.g., geographic location, professional affiliation).

**Results:**

Retracted articles related to COVID-19 were shared tens of thousands of times to an audience of potentially hundreds of millions of readers and followers. Twitter was the largest medium for sharing these articles, and the United States was the country with the most sharers. While general members of the public were the largest proportion of sharers, researchers and professionals were not immune to sharing these articles on social media and on websites, blogs, or news media.

**Conclusions:**

These findings have potential implications for better understanding the spread of misleading or false information perpetuated in retracted scholarly publications. They emphasize the importance of quality peer review and research ethics among journals and responsibility among individuals who wish to share research findings.

## INTRODUCTION

The COVID-19 pandemic remains a serious health concern worldwide despite vaccine development. This emergency situation resulted in a vast body of literature related to the pandemic. Since February 20, 2020, 137 scientific papers have been published per day, on average, on the topic of COVID-19 [1]. Universal hurry related to the production of scholarship on the pandemic has increased the speed of scientific productivity and researchers' inaccuracy and has accelerated the review process, inviting a greater likelihood of research misbehavior [2]. Thus, the rapid growth of COVID-19 literature has led to a major, unfortunate outcome; the ratio of retracted articles is much greater, compared historically, than that for other research topics [1].

Scientific misconduct plays a prominent role in the retraction of articles from the biomedical literature [3]. Scientific misconduct is “fabrication, falsification or plagiarism in proposing, performing or reviewing research or in reporting research results” according to the US Office of Research Integrity [4]. Serious deficiencies in the quality of empirical findings and conclusions drawn by these articles [5] could undermine scholars' trust in their reported results [2]. Furthermore, retractions can have negative consequences for authors [6] and lead to irrecoverable injuries for patients if improper methodologies or findings from these retracted studies are employed in clinical settings [7].

During the COVID-19 pandemic, with most individuals practicing social distancing and isolating to protect themselves, online sources were increasingly consulted in order to find and share information [8,9]. Social platforms, including social media and research sharing websites (e.g., Mendeley), have the tremendous capability to increase the visibility of and disseminate coronavirus-related research [10,11]. However, invalid or distorted information shared on social platforms can have serious adverse effects on readers as well as medical practitioners, who may use information in retracted articles to inform clinical decision-making [12,13,14].

Altmetrics are alternative metrics to traditional bibliometric indicators (e.g., citations) that document the impact or dissemination of scholarly works on platforms like social media, online news sites, blogs, and research-sharing sites. Altmetrics are a rapid indicator of the impact of an article within public discourse, as the article can be “cited” on platforms like Twitter on the same day it is published [15]. Recent studies show that retracted articles published in reputable journals, like *Lancet* or *New England Journal of Medicine,* can attain “astronomical” altmetrics scores through being spread on social platforms, especially Twitter [16]. Given the importance of social media to the dissemination of COVID-19 information, some altmetrics studies have already been performed, but they lack a specific and extensive focus on retracted articles [17–21].

Given the potentially dangerous impact of retracted COVID-19 articles on disease prevention and intervention and the unique role of social media and other article-sharing platforms in this era, this study aims to expand on a prior study by Cortegiani et al. [13] to provide insight into the current state of retracted COVID-19 articles through an altmetrics lens. While Cortegiani et al.'s study focused on social media coverage of retracted articles in general, we focus on altmetrics for retracted COVID-19 articles specifically. Our central research question is to what extent have retracted COVID-19 articles been shared online prior to retraction as indicated by altmetrics? In particular, we sought to determine how many times these articles were shared, who was responsible for sharing them, and the potential size of the audience.

## METHODS

Data for this study were retrieved in early March 2021 from three sources: the Retraction Watch Database (https://retractiondatabase.org/), Scopus, and PubMed. Using an approach adapted from Soltani and Patini's 2020 study of retracted COVID-19 articles, the following search terms were used to identify retracted manuscripts originally published between January 1, 2020, and March 1, 2021: “COVID-19,” “coronavirus disease 2019,” “coronavirus 2019,” “coronavirus 2020,” “SARS-COV-2,” and “2019-nC0V.” Only articles that were retracted based on a factual or methodological error on the part of the author were included; articles that were retracted only due to a publisher error were not included. Note, however, that over time some articles may be revised and republished following their retraction, which may mean that some articles classified as retracted in this study may have been republished at the time of this writing.

Seventy-four retracted articles were initially retrieved using this strategy. However, three of these articles lacked a digital object identifier (DOI) or PubMed ID, which made it impossible to collect altmetric data, and were thus excluded from the study. Of the remaining seventy-one articles, thirty-nine had an Altmetric Attention Score, whereas thirty-two were not shared before retraction. These thirty-nine articles constituted the final population for this study (Appendix A).

Altmetric Attention Score for each article was obtained from the Altmetrics Bookmarklet, available at https://www.altmetric.com, on March 1, 2021. This application aggregates sharing data for all social platforms, including how frequently an article has been shared on each platform and potential audience size (i.e., number of subscribers to sharers' accounts). These data are aggregated from a wide variety of platforms, including public policy documents (particularly relevant during the COVID-19 pandemic), online publication-sharing platforms (e.g., Mendeley, ResearchGate), Wikipedia, blogs, social media sites (e.g., Facebook, Twitter, Reddit, Weibo), and video-sharing platforms (e.g., YouTube).

As Altmetric Attention Scores are updated each time an article is shared, scores collected for analysis are reflective only of a particular point in time; however, collection of altmetrics data for this study was performed after all articles had been retracted. Among the 39 articles examined, a mean of 203 days had passed from the date of retraction to the date of data collection (median = 227 days), with a range of 10 to 378 days. The correlation between the time since retraction and Altmetric Attention Score was 0.19, indicating that articles retracted further in the past did not necessarily accrue higher scores.

Using the Altmetrics Bookmarklet, an altmetrics page can be accessed for each article included in the Altmetrics database, which includes the overall Altmetric Attention Score and a breakdown of its contributing sources. By clicking on a section entitled “click for more details,” a more detailed breakdown is shown, including profile information for individuals who shared an article on social media or saved the article on Mendeley. This allows the reader or researcher to have a better understanding of not only how much an article has been shared, saved, or discussed but also who has done the sharing, saving, or discussing. For the disciplinary affiliation (e.g., medical sciences, natural sciences, social sciences) of Mendeley readers, information from user profiles was used to thematically align readers with the most appropriate Mendeley discipline classification. In some cases, this process is nearly automatic, based on data provided in the Altmetrics Bookmarklet, but in other cases it requires careful inspection of a profile and agreement among the researchers as to the most appropriate affiliation.

Data were transferred from the Altmetrics Bookmarklet to Microsoft Excel for further analysis. While a single member of the research team was responsible for initially collecting the data, all data were checked by a second team member using the Bookmarklet and Excel spreadsheet to ensure accuracy in transcribing the scores.

## RESULTS

### Platforms contributing to Altmetric Attention Scores

**Figure 1:**
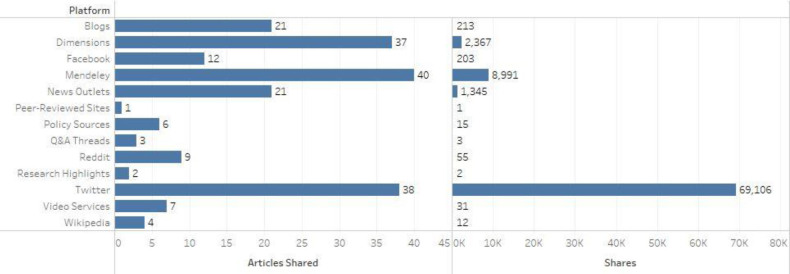
Number of articles shared and total shares on each platform

The number of articles shared or saved on each platform and the total number of shares for each are shown in Figure 1. The most prominent platforms were Mendeley, which registers when a user saves an article to their account, and Twitter, which registers when a user tweets about an article. The fact that these platforms are responsible for the most shares is logical, given that the effort to share an article on them is as simple as saving an article or tweeting or retweeting about it. Conversely, registering a “share” on Wikipedia or in a policy source requires considerable effort, as the author must actually write or edit an article or policy that cites one of the retracted articles.

### Demographics of sharers of retracted articles

The geographic distribution of Twitter sharers based on information in their account profiles and geotagging of their tweets provides an idea of which countries were most responsible for sharing these retracted articles. As shown in Table 1, the largest percentage of tweets originated in the United States, United Kingdom, France, and Japan.

**Table 1:** Number of tweets about retracted articles by country

Country	Number of tweets	Percentage of tweets
United States	8,434	16.00%
United Kingdom	4,382	8.31%
France	4,007	7.60%
Japan	2,147	4.07%
Brazil	1,443	2.74%
Canada	1,163	2.21%
Spain	882	1.67%
Thailand	736	1.40%
Mexico	400	0.76%
Germany	322	0.61%
El Salvador	260	0.49%
Australia	241	0.46%
Italy	139	0.26%
Ukraine	89	0.17%
Colombia	86	0.16%
Korea, Republic of	85	0.16%
Hong Kong	85	0.16%
China	84	0.16%
Netherlands	80	0.15%
India	73	0.14%
Poland	41	0.11%
Chile	18	0.03%
Korea, Democratic People's Republic of	11	0.02%
Switzerland	7	0.01%
Romania	4	0.01%
Solomon Islands	4	0.01%
Nepal	3	0.01%
Singapore	3	0.01%
Ecuador	3	0.01%
Ireland	3	0.01%
Turkey	2	0.01%
Argentina	2	0.01%
South Africa	2	0.01%
Costa Rica	2	0.01%
Kenya	2	0.01%
Sweden	1	0.01%
New Zealand	1	0.01%
Bermuda	1	0.01%
Comoros	1	0.01%
Indonesia	1	0.01%
Curagao	1	0.01%
Sierra Leon	1	0.01%
Iceland	1	0.01%
Saudi Arabia	1	0.01%
Unknown	27,456	51.94%

Table 2 displays the demographic breakdown of individuals who tweeted about the retracted articles. Members of the general public were the most common Twitter users, rather than scientists, doctors, or writers. However, substantial portions of Twitter users were members of one of these three professional groups, who have important roles in vetting the quality of and disseminating scientific findings to the public.

**Table 2:** Demographic breakdown of Twitter users

Rank	Twitter users	Number of tweets	Percentage of tweets
1	Members of the public	64,810	94.2%
2	Scientists	2,284	3.3%
3	Practitioners (doctors, other health care professionals)	1,033	1.5%
4	Science communicators (journalists, bloggers, editors)	608	0.9%

Table 3 shows the backgrounds of readers of the retracted articles in Mendeley. Fourteen percent of all Mendeley readers were professional researchers. Students, though, represented the largest group of identified readers, with bachelor's, master's, and PhD/doctoral student readers representing a combined 31%.

**Table 3:** Demographic breakdown of Mendeley readers

Rank	Readers by professional status	Number of readers	Percentage of readers
1	Researcher	1,247	14%
2	Bachelor's student	1,002	11.3%
3	Master's student	926	10.4%
4	PhD student	683	7.7%
5	Doctoral student	117	1.3%
6	Lecturer	31	0.4%
7	Postgraduate student	29	0.3%
8	Librarian	12	0.1%
9	Professor	10	0.1%
10	Associate professor	10	0.1%
12	Unknown/unspecified/other	4,823	54%

### Discipline of readers of retracted articles

Table 4 displays the disciplinary affiliations of readers who saved the retracted articles on Mendeley. Unsurprisingly, a large portion of readers whose affiliation could be identified were associated with a biomedical discipline (∼82%). Comparatively, social sciences disciplines had very little representation among Mendeley readers (∼3%).

**Table 4:** Discipline of Mendeley readers

Rank	Discipline	Number of readers	Percentage
1	Medicine & dentistry	2,517	28.8%
2	Biochemistry, genetics & molecular biology	503	5.8%
3	Nursing & health professions	415	4.8%
4	Agricultural & biological sciences	341	3.9%
5	Pharmacology, toxicology & pharmaceutical science	205	2.4%
6	Immunology & microbiology	180	2.1%
7	Engineering	157	1.8%
8	Computer science	140	1.6%
9	Psychology	66	0.8%
10	Social sciences	47	0.5%
11	Environmental science	47	0.5%
12	Neurosciences	16	0.2%
13	Earth & planetary sciences	9	0.1%
14	Business, management & accounting	7	0.1%
15	Chemistry	3	0.03%
16	Philosophy	1	0.01%
17	Economics, econometrics & finance	1	0.01%
18	Arts & humanities	1	0.01%
19	Material Sciences	1	0.01%
20	Chemical engineering	1	0.01%
21	Unknown/unspecified/other	4,076	46.5%

## DISCUSSION

This study indicates that retracted COVID-19 articles have been widely disseminated and shared online as evidenced by Altmetric Attention Scores. Twitter, in particular, is the platform with the widest sharing of retracted articles, with nearly 70,000 tweets reaching an audience of potentially over 50 million followers, which could cause substantive damage if the retracted articles disseminate false or misleading information. Furthermore, news outlets, which tend to have a significant audience and reach, shared the articles or information over 1,000 times, illustrating the breadth with which misinformation may be spread.

Members of the general public (i.e., non-scientists/experts) appeared to be the most common sharers of retracted articles, although most sharers on Mendeley and other scholarly platforms were professionals or scholars. Among these professionals, those in biomedical fields appeared to be most likely to share. This is potentially problematic, as medical professionals often use recent scholarly research to inform clinical decision-making, such as diagnoses, treatment, and other medical advice offered to patients. Retracted medical papers could lead to deleterious health and medical decisions, as seen in the wake of studies in the early twenty-first century that linked vaccines with autism [21].

Libraries, particularly those that specialize in medical sciences or work frequently with the public, can play an important role in educating about COVID misinformation [22,23]. While it may not be possible or reasonable to expect librarians to identify or be aware of all problematic articles that are circulating on the Internet, awareness of the extent to which retracted COVID-19 articles have been circulated could be beneficial when assisting patrons in parsing through emerging literature. Resources like Retraction Watch can be used to inform inquiring members of the public about these articles' statuses.

The main limitation of this study relates to limitations on data availability. Particularly with Mendeley, professional affiliations and research areas were not available for approximately half of researchers. Though it may be possible to match researchers through further searching (e.g., checking university websites), the time required to find this information is prohibitive. Further research could also expand this analysis by examining the context of how these retracted articles are cited in social media and other platforms. For instance, it is possible that some sharers may have shared an article with a note like “great information!” or “I'm not sure about this one,” with each conveying a different tone about the article. Additionally, our study's scope was limited to English-language articles, as we were not able to easily analyze articles published in other languages.

In conclusion, the sharing of retracted articles related to COVID-19 has created a pandemic of its own—one of the rapid spread of incorrect or misleading facts, or infodemic. This study demonstrates the prodigious reach of only thirty-nine retracted COVID-19 articles over a short time frame. The impact of this spread, affecting potentially tens of millions of people, is difficult to comprehend and illustrates the irreparable harm that can be done when rigorous peer review and research ethics are not observed.

## Data Availability

All data associated with this article are available at http://dx.doi.org/10.13140/RG.2.2.36469.70885.

## SUPPLEMENTAL FILES

**Appendix A.** Articles included in this study
