# Supplementary material for: Sharing of retracted COVID-19 articles: an altmetric study
Source: J Med Libr Assoc. 2022 Jan 1;110(1):97–102. doi: 10.5195/jmla.2022.1269 (PMC8830369; doi:10.5195/jmla.2022.1269)
Supplement: Supplementary file 1 — Appendix A. Articles included in this study [file jmla-110-1-97-s01.docx]

**Appendix A. Articles Included in This Study**

| Title | Status | Retraction date and time passed before this study | Journal | Altmetrics score |
| --- | --- | --- | --- | --- |
| Hydroxychloroquine or chloroquine with or without a macrolide for treatment of COVID-19: a multinational registry analysis | Retracted | 06/14/2020  260 days | *The Lancet* | 23168 |
| Effectiveness of Surgical and Cotton Masks in Blocking SARS-CoV-2: A Controlled Comparison in 4 Patients | Retracted | 06/02/2020  272 days | *Annals of Internal Medicine* | 7164 |
| Cardiovascular Disease, Drug Therapy, and Mortality in Covid-19 | Retracted | 06/04/2020  270 days | *New England Journal of Medicine* | 3802 |
| Potential false-positive rate among the ‘asymptomatic infected individuals’ in close contacts of COVID-19 patients | Withdrawn | 03/09/2020  363 days | *Zhonghua Liu Xing Bing Xue Za Zhi* | 3733 |
| 5G Technology and induction of coronavirus in skin cells | Retracted | 07/16/2020  227 days | *Journal of biological regulators and homeostatic agents* | 3328 |
| SARS-CoV-2 infects T lymphocytes through its spike protein-mediated membrane fusion | Retracted | 06/15/2020  259 days | *Cellular and Molecular Immunology* | 3196 |
| Chinese medical staff request international medical assistance in fighting against COVID-19 | Retracted | 02/26/2020  369 days | *The Lancet* | 2916 |
| Chloroquine or hydroxychloroquine for COVID-19: why might they be hazardous | Retracted | 7/09/2020  234 days | *The Lancet* | 1319 |
| Can Traditional Chinese Medicine provide insights into controlling the COVID-19 pandemic: Serpentinization-induced lithospheric long-wavelength magnetic anomalies in Proterozoic bedrocks in a weakened geomagnetic field mediate the aberrant transformation of biogenic molecules in COVID-19 via magnetic catalysis | Withdrawn | 11/05/2020  108 days | *Science of The Total Environment* | 1214 |
| mRNA Vaccines to Prevent COVID-19 Disease and Reported Allergic Reactions: Current Evidence and Suggested Approach | Removed | 12/31/2020  59 days | *The Journal of Allergy and Clinical Immunology* | 938 |
| Clinical and epidemiological characteristics of 34 children with 2019 novel coronavirus infection in Shenzhen | Retracted | 02/17/2020  378 days | *Chinese journal of pediatrics* | 567 |
| Homeopathy combat against coronavirus disease (Covid-19) | Retracted | 08/03/2020  208 days | *Journal of Public Health* | 482 |
| Mortality of a pregnant patient diagnosed with COVID-19: A case report with clinical, radiological, and histopathological findings | Withdrawn | 06/01/2020  273 days | *Travel Medicine and Infectious Disease* | 356 |
| Obesity and mortality of COVID-19. Meta-analysis | Retracted | 02/06/2021  21 days | *Obesity Research & Clinical Practice* | 160 |
| Clinical manifestations and outcome of SARS-CoV-2 infection during pregnancy | Withdrawn | 01/31/2021  27 days | *Journal of Infection* | 85 |
| Corona Virus Killed by Sound Vibrations Produced by Thali or Ghanti: A Potential Hypothesis | Removed | 06/01/2020  272 days | *Journal of Molecular Pharmaceuticals and Regulatory Affairs* | 50 |
| Mental health burden for the public affected by the COVID-19 outbreak in China: Who will be the high-risk group? | Retracted | 10/23/2020  122 days | *Psychological Health Medicine* | 40 |
| No deleterious effect of lockdown due to COVID-19 pandemic on glycaemic control, measured by glucose monitoring, in adults with type 1 diabetes | Retracted | 07/27/2020  215 days | *Diabetes Technology Therapy* | 27 |
| Clinical sequelae of the novel coronavirus: Does COVID-19 infection predispose patients to cancer? | Retracted | 12/02/2020  88 days | *Future Oncology* | 25 |
| Calcifediol treatment and COVID-19-related outcomes | Removed | 02/19/2021  10 days | *Preprints with the Lancet* | 21 |
| Lung ultrasound score in establishing the timing of intubation in COVID-19 interstitial pneumonia: A preliminary retrospective observational study | Retracted | 11/30/2020  90 days | *PLoS One* | 19 |
| Chinese mental health burden during the COVID-19 pandemic | Retracted | 09/03/2020  177 days | *Asian Journal of Psychiatry* | 15 |
| COVID-19 in Africa and collateral effects on health systems and their immunization programs | Withdrawn | 12/28/2020  62 days | *Vaccine* | 13 |
| Molecular Phylogeny and Revision of Copepod Orders (Crustacea: Copepoda) | Retracted | 10/14/2020  130 days | *Scientific Reports* | 13 |
| Incidence and mortality of COVID-19 in Iranian multiple sclerosis patients treated with disease-modifying therapies | Withdrawn | 10/08/2020  136 days | *Revue Neurologique* | 11 |
| Managing college operations during the coronavirus outbreak | Withdrawn | 04/10/2020  325 days | *Journal of the American Pharmacists Association* | 5 |
| Nurses reports of actual work hours and preferred work hours per shift among frontline nurses during coronavirus disease 2019 (COVID-19) epidemic: A cross-sectional survey | Withdrawn | 05/16/2020  309 days | *International Journal of Nursing Studies* | 5 |
| Effects of the COVID-19 Pandemic on Stroke Patients | Retracted | 01/13/2021  45 days | *Cureus* | 4 |
| Resistance to Nano-Based Antifungals Is Mediated by Biomolecule Coronas | Retracted | 03/24/2020  342 days | *ACS Applied Materials and Interfaces* | 3 |
| Mechanical ventilation in COVID-19: Is it due to patient or virology factors? | Withdrawn | 06/14/2020  260 days | *Annals of Medicine and Surgery* | 2 |
| Liver impairment associated with disease progression in COVID-19 patients | Withdrawn | 09/01/2020  179 days | *Liver International* | 2 |
| From SARS-CoV to Wuhan 2019-nCoV Outbreak: Similarity of Early Epidemic and Prediction of Future Trends | Withdrawn | 01/28/2020  398 days | *bioRxiv* | 2 |
| Deep learning system to screen coronavirus disease 2019 pneumonia | Retracted | 04/22/2020  312 days | *Applied Intelligence* | 1 |
| Phytotherapeutic options for the treatment of COVID-19: A concise viewpoint | Retracted | 12/30/2020  61 days | *Phytotherapy Research* | 1 |
| A mechanistic analysis placental intravascular thrombus formation in COVID-19 patients | Withdrawn | 06/22/2020  258 days | *Annals of Diagnostic Pathology* | 1 |
| Selenium – associated gene signatures within the SARS-CoV-2 – host genomic interaction interface | Withdrawn | 07/15/2020  228 days | *Free Radical Biology and Medicine* | 1 |
| Analysis of thin-section CT in patients with coronavirus disease (COVID-19) after hospital discharge | Withdrawn | 05/15/2020  310 days | *Clinical Imaging* | 1 |
| NY-SAR-35 is involved in apoptosis, cell migration, invasion and epithelial to mesenchymal transition in glioma | Retracted | 02/17/2020  378 days | *Biomedicine & Pharmacotherapy* | 1 |
| Globalization and vulnerable populations in times of a pandemic: A Mayan perspective | Retracted | 12/14/2020  77 days | *Philosophy, Ethics, and Humanities in Medicine* | 1 |
